# Secondary Repair of Jersey Finger: A Novel Method of Tendon Length Estimation via Measurement of Adjacent Landmarks

**DOI:** 10.7759/cureus.53963

**Published:** 2024-02-10

**Authors:** Arjan Singh Sehmbi, Mobin Syed, Aashtad Cyrus Daruwalla, Peter Rae, Isaac Harris, Savan Shah, David Parry

**Affiliations:** 1 Trauma and Orthopaedics, University College Hospital, London, GBR; 2 Anatomy, King's College School of Medicine, Guy's and St Thomas' NHS Foundation Trust, London, GBR; 3 Otolaryngology, Sheffield Teaching Hospitals NHS Foundation Trust, Sheffield, GBR; 4 Faculty of Life Sciences and Medicine, King's College London, London, GBR

**Keywords:** fdp avulsion, secondary repair, tendon avulsion rupture, flexor digitorum profundus tendon, jersey finger

## Abstract

Jersey finger describes the rupture of the flexor digitorum profundus (FDP) tendon at its insertion into the distal phalanx. In the absence of an evidence-based approach to tensioning during secondary repair, we aimed to devise a novel method to determine the required tendon length pre/intraoperatively. We measured anatomical landmarks, associated with the FDP tendon, on dissected cadavers, to assess whether these can be used to estimate tendon segment lengths. Eight cadaveric hands were dissected. Three measurements from the distal lumbrical origin to (1) FDP insertion, (2) the distal end of A1 (Annular 1 pulley), and (3) the proximal end of A1 were recorded for digits II-V.

Relative ratios for measurement 1 were consistent for all digits, compared to digit III. Linear regression analysis confirmed a strong correlation for measurement 1 between digit II (R^2 ^=0.97) and digit IV(R^2 ^=0.97) compared to digit III across all specimens. Digit III distal lumbrical origin to FDP insertion measurements could facilitate the estimation of the required graft length for digit II or IV during secondary repair.

This is a level IV study, providing proof of concept for a novel method of tendon tensioning.

## Introduction

A ‘jersey finger’ injury is characterised by the rupture of the flexor digitorum profundus (FDP) tendon at its insertion into the distal phalanx. It typically presents as a sporting injury when one player grasps an opponent’s jersey with their fingertip, resulting in forced hyperextension in active flexion [1]. While these injuries can affect individuals of all ages, football, rugby and rock climbing are the most commonly implicated activities [2]. While sports are the most common setting for jersey finger injuries, similar mechanisms of injury can occur in occupational settings, such as construction or manual labour, where fingers may be caught or trapped in machinery or equipment. The fourth digit accounts for 75% of cases [3]. This is likely due to the fourth digit being approximately 5 mm longer than the other digits when flexed at the proximal interphalangeal joint (PIPJ) [4]. An audible ‘pop sound’, followed by impaired flexion, localised tenderness and swelling is often experienced. The affected finger remains extended compared to the others in the relaxed position [5]. Minimal pain and functional loss often result in a delayed presentation; Gaston et al. found that only half of cases present acutely [6].

Clinical diagnosis

The sweater finger sign can be elicited to facilitate clinical diagnosis. This describes an inability to flex the distal interphalangeal joint (DIPJ) with the PIPJ held in extension in the affected finger [7]. Plain radiographs can identify associated avulsed fracture fragments whilst magnetic resonance imaging (MRI) can visualise the ruptured tendon segment [8].

Management options

Early surgical repair is recommended to optimise functional outcomes. The choice of primary or secondary repair is dictated by the time from injury to presentation and the severity of the injury. Associated vincula rupture can compromise vascular supply and tendon nutrition, resulting in fibrosis. Early surgical intervention is crucial in such cases. Primary repair is preferred in cases presenting acutely and involves reattachment of the tendon/avulsed bone segment to the distal phalanx. Other options exist for cases presenting sub-acutely or chronically including conservative (if function is preserved), DIPJ arthrodesis or secondary tendon grafting. The latter of these is favoured for reconstruction to achieve acceptable functional outcomes. Repair can be single or two-staged. The ipsilateral palmaris longus or plantaris tendon is harvested in a single or two-stage repair, the former of which relies on an intact tendon sheath and fibro-osseous tunnel [9].

Aims

A common obstacle encountered during secondary repair is the degree of tension under which to place the graft. Under-tensioning causes asynchronous and inefficient flexion whilst over-tensioning results in a fixed flexion deformity and a quadriga phenomenon. This describes reduced flexion in the adjacent non-injured fingers due to a shortened FDP tendon in the affected digit [10]. In the absence of an evidence-based approach to this problem, we aim to use cadaveric dissection data to devise a novel technique of measuring adjacent landmarks to enable surgeons to estimate the required graft length pre/intra-operatively. This should facilitate junior surgeons to avoid tensioning-related complications post-operatively.

Ethics

All dissections were carried out on donors under the consent of the Human Tissue Authority, under license number 12123 at King’s College London.

## Materials and methods

Cadaveric dissection

**Figure 1:**
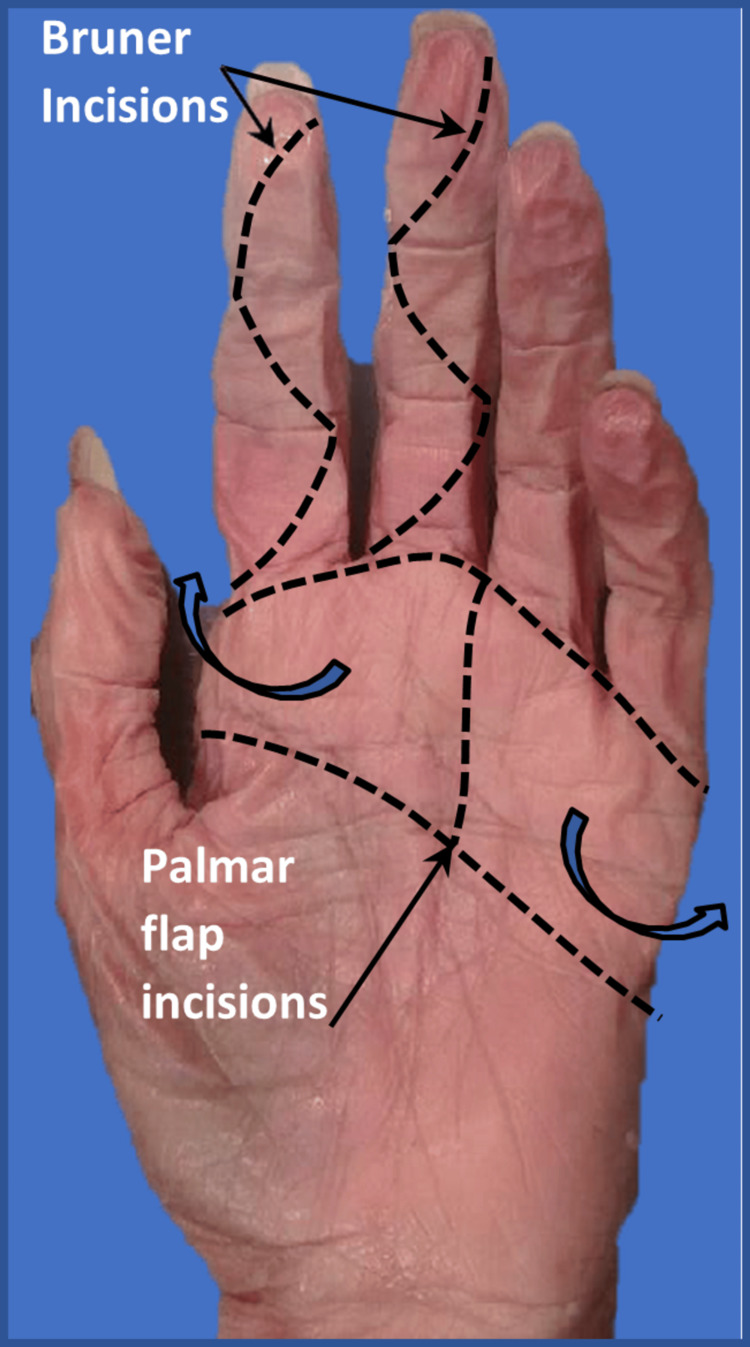
Palmar and digital incisions made during dissection

**Figure 2:**
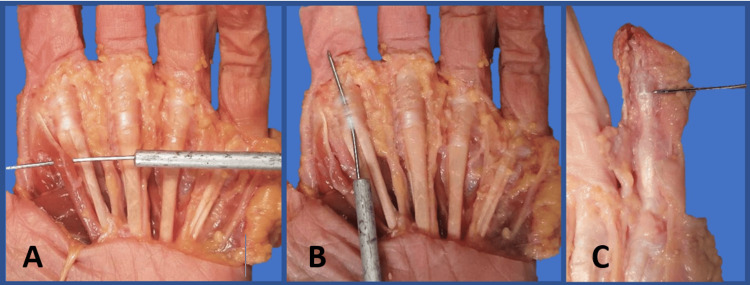
Key anatomical landmarks identified during dissection A: Lumbrical muscle to digit II; B: A1 pulley over digit II; C: FDP tendon distal insertion in digit V

Eight cadaveric upper limbs were dissected; four were formalin-fixed and four were non-embalmed. Dissections and images taken were in accordance with the Human Tissue Act 2004. A proximal transverse incision was made across the volar aspect of the hand, in line with the proximal transverse skin creases of the palm. A second distal transverse incision was made across the volar surface of the proximal end of the digits, followed by a longitudinal incision traversing these two incisions between digits III and IV (Figure 1). The underlying palmar aponeurosis was incised, with the remaining fat removed using blunt dissection. This allowed visualisation of the flexor digitorum superficialis (FDS) tendon, the FDP tendon, and the insertion of the lumbrical muscles into the FDP tendons (Figure 2A). A dissecting needle was passed along the tendons into the fibro-osseous tunnel to identify the proximal and distal aspects of the A1 pulley (Figure 2B). Bruner-type incisions were made longitudinally along the full length of each digit II-V, exposing the tendons and surrounding pulley system from the proximal to the distal end [11]. Dissection of the distal phalanx allowed the identification of the insertion point of the FDP tendon into bone (Figure 2C).

Length measurement protocol

**Figure 3:**
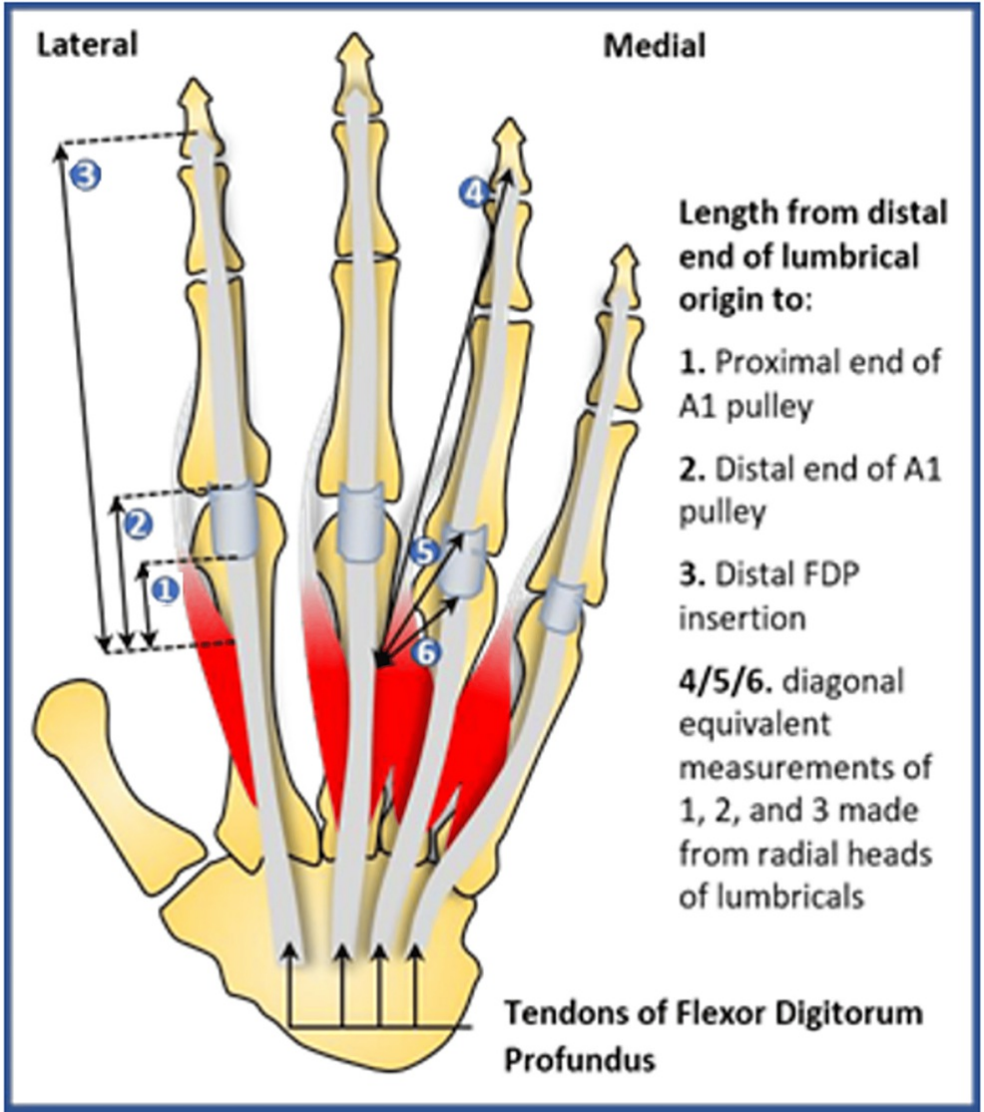
Measurements taken illustrated on the anterior surface of the left hand Image credits: Dr. Arjan S Sehmbi

We used the following length measurements: the distal end of the lumbrical origin to the distal insertion of the FDP, the distal end of A1 and the proximal end of A1 (measurements 1, 2 and 3, respectively, in Figure 3). Measurements were taken using a 0.1 mm screw gauge, accurate to ±0.02 mm. Measurements were taken with the hand fixed flat, volar side up, and each digit in the neutral position. Points of measurement were verified by two researchers, independently. The bipennate nature of the lateral two lumbricals resulted in two measured lengths for digit IV and V for each of the points. Measurements from the radial heads of the lumbricals were made in a diagonal plane to the FDP tendon (in addition to the vertical measurement) as illustrated in Figure 3 by lengths 4-6. These landmarks were chosen due to the minimal anatomical variability with respect to the lumbrical insertion [12]. Standard deviation and paired t-test were calculated for each measurement in Figure 3 to assess the variance in collected data.

## Results

Cadaver demographics

The four formalin-fixed hands came from two embalmed bodies and consisted of two right (50%) and two Left (50%) hands. All formalin fixed cadavers were female. The four non-embalmed hands originated from four separate donors and comprised three left hands (75%) and one right hand (25%). The sex of these hands used was unknown, as they were supplied separately. Of the eight cadaveric samples, five were left hands (62.5%) and three were right (37.5%). There were no significant anatomical variations or pathologies present that hindered the dissection process or collected data.

Lumbrical relations

All measurements were made between the distal lumbrical origin to the landmarks outlined in Figure 3. Variability in hand size meant the analysis of absolute values was of less value than the analysis of the ratios between the measurements. Digit III measurements were selected to be the basis of comparison for the other digits (II, IV and V). Only the ulna head values were used for bipennate lumbricals, as diagonal measures made from radial heads are not comparable to the parallel measures from ulna heads (Figure 3). The ratio between these two points was calculated for each of these three digits compared to digit III by dividing the given digit’s measured length by digit III’s measured length. Therefore, relative ratio = Digit x measured length/digit III measured length. The mean relative ratios for each of the digits for all three measurements taken are presented in Table 1. Standard deviation (σ) values were calculated to determine whether the variance of the relative ratio measurements for each digit was too high to accurately utilise the mean. Table 1 demonstrates a greater than 10-fold difference in σ between measurement 1 as compared to measurements 2 and 3. This would suggest that the use of relative ratios to estimate lengths involving the A1 pulley system is inaccurate. Conversely, σ values for measurements to the FDP insertion were relatively low, ranging between 0.021-0.049, indicating minimal variance in relative ratios and an increased probability of the calculated mean representing the population. Paired t-tests verified this by showing a significantly lower spread of data for relative ratios calculated for measurement 1 compared to measurement 2 (p < .00001) and measurement 3 (p < .00001).

**Table 1:** Relative ratios of measurement 1, 2 and 3 at each digit compared to digit III Lengths 1: Length from the distal end of lumbrical origin to the proximal end of A1 pulley; Lengths 2: Length from the distal end of lumbrical origin to the distal end of A1 pulley; Lengths 3: Length from the distal end of lumbrical origin to distal FDP insertion Statistical test used: mean and standard deviation (SD) of relative ratio

Digit	Relative ratio of length 1 in digit x compared with III		Relative ratio of length 2 in digit x compared with III		Relative ratio of length 3 in digit x compared with III	
	Mean	SD	Mean	SD	Mean	SD
II	0.878	0.021	1.015	0.308	1.029	0.225
III	1	0	1	0	1	0
IV (radial head of lumbrical)	0.951	0.0465	1.101	0.261	1.326	0.494
IV (ulnar head of lumbrical)	0.940	0.0354	0.981	0.159	1.017	0.174
V (radial head of lumbrical)	0.734	0.050	0.935	0.207	0.970	0.377
V (ulnar head of lumbrical)	0.742	0.045	0.956	0.184	0.899	0.236

**Figure 4:**
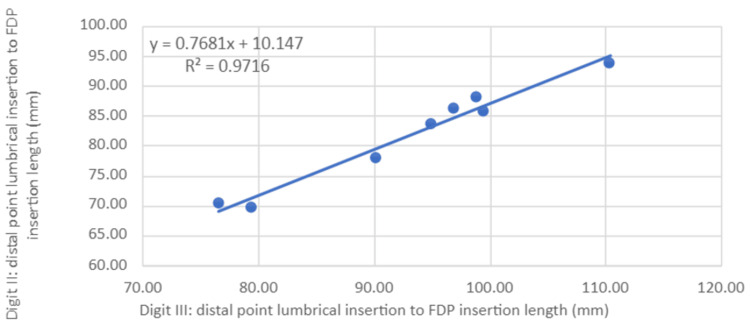
Ratio of distal point of lumbrical origin to FDP insertion length between digits III and II Statistical test: linear regression (y) and coefficient of determination (R2) FDP: flexor digitorum profundus

**Figure 5:**
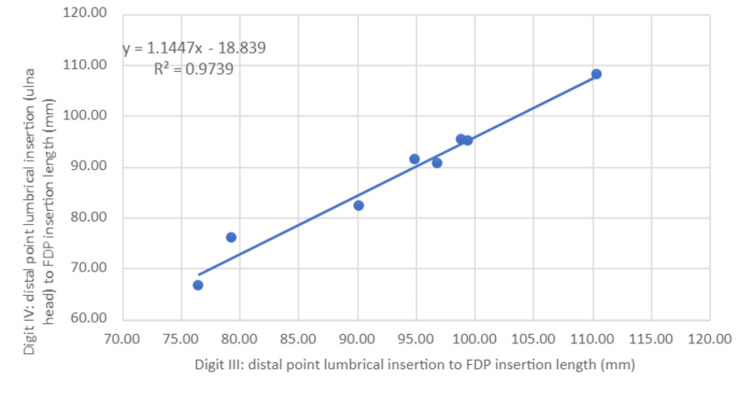
Ratio of distal point lumbrical origin to the FDP insertion length between digits III and IV Statistical test: linear regression (y) and coefficient of determination (R^2^) FDP: flexor digitorum profundus

**Figure 6:**
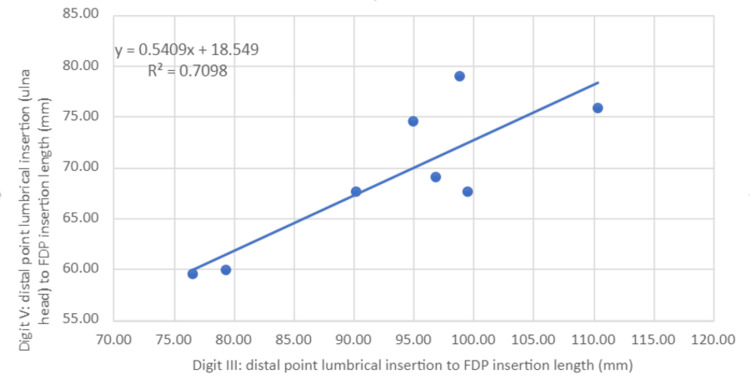
Ratio of distal point lumbrical origin to the FDP insertion length between digits III and V Statistical test: Linear regression (y) and coefficient of determination (R2)

Scatter plots for measurement 1 were then generated (Figures 4-6) with a line of best fit included using linear regression analysis for digits II, IV and V compared to III. R2 values for both digits II and IV were over 0.97 (III and II = 0.972, III and IV = 0.974), indicating high predictive accuracy whilst digit V had a relatively lower R2 (III and V = 0.71).

Worked clinical example

A patient presents two months after a sporting injury to the right hand and is diagnosed clinically with a jersey finger at digit IV. The decision to proceed with secondary repair using a two-stage tendon repair is based on clinical presentation and functional ability. Preoperative MRI revealed measurement 1 for digit III as 100.00 mm. Our linear regression model predicts that measurement 1 for digit IV should be 95.63 mm by plotting the measurements on the linear regression graphs (Figure 5). Therefore, the surgeon can ensure measurement 1 at digit IV is equal to the predicted value intra-operatively when attaching the graft tendon. This therefore acts as an aid in achieving adequate tensioning intraoperatively.

## Discussion

We found that the distal lumbrical origin to the FDP insertion measurement can be used to estimate the length of graft length required in digit II or IV using measurements from digit III. During the measurement process, we found that defining the measurement points was more difficult for the medial two digits due to the bipennate nature of the associated lumbricals and smaller size muscles and pulleys. This resulted in greater variability in relative ratios for these two digits as seen in Table 1. We also found that the relative ratio measurement involving measurements 2 and 3 showed greater variability compared to those involving measurement 1. This may be due to difficulty in the identification of the A1 pulley boundaries (particularly distally) as well as the established anatomical variance in A1 pulley length as reported by Fiorini et al. [13]. All relative ratios for measurement 1 were <1 when compared to II, as would be expected. There were also statistically significant lower σ values for measurement 1 compared to measurements 2 and 3 (Table 1). This may be due to the FDP insertion point being easier to define, thereby resulting in greater accuracy in measurement [14].

The above findings helped narrow our focus on measurements involving measurement 1. Linear regression analyses provided an additional means of defining the predictive value between two data sets as displayed in Figures 4-6. The high predictive value for measurement 1 in digits II and IV compared to III indicates a high likelihood of accurately estimating measurement 1 lengths in these digits when provided with the same measurement 1 length in digit III.

To our knowledge, this study is the first of its kind to propose a standardised approach to this commonly encountered issue. Implementation of our methodology has the potential to facilitate pre-operative MRI-assisted measurement of adjacent landmarks to estimate the required graft length. This will allow surgeons to mitigate tensioning-related issues which impact the outcome of the procedure and therefore the function of the hand.

Limitations

Although all measurements were verified by two researchers independently, observer-reliant data is always vulnerable to human error. The small sample size in our study limits the generalisability of our conclusions. However, this study does provide a proof of concept that can be expanded on with additional testing. Both non-embalmed and formalin-fixed cadavers were analysed as a whole data set. Although they followed the same trend in our limited sample size, they may show differences at a larger scale and warrant investigating separately. Brenner (2014) found that formalin-fixed specimens have altered biomechanical properties, which may influence the validity of our results [15]. Not all centres currently perform pre-operative MRI or specialist ultrasound scan (USS) for the assessment of closed FDP ruptures. Our study proposes the use of such imaging for operative planning in cases of delayed repair of FDP rupture. The results from the cadaveric study will help obtain the final predicted length of the tendon for the index and ring fingers. However, additional tendon length might be required in practice to allow for the tendon to be used for part of the repair. Owing to the challenges of identifying the lumbrical origin and point of insertion of FDP, collaboration with specialist musculoskeletal system (MSK) radiologists will likely be needed to determine the length of measurement 1. The current system of relative ratios and linear regression is based on taking digit III as the basis. This means we are unable to estimate middle finger tendon segment lengths. However, in clinical practice, this may not be a significant obstacle, as the majority (75%) of jersey finger cases affect digit IV [3]. The graft length required by the surgeon may vary from the length we are able to predict using the current methodology based on the clinical scenario. For instance, if the required length extends proximal to the lumbrical origin, the presently proposed model will be ineffective in calculating the total length but will still provide the required length between the landmarks. In our study, measurements were all taken with the hands flat and digits in the neutral position. In a clinical setting, such as during pre-operative imaging or intraoperatively, achieving and maintaining this position may be more challenging. If the hand cannot be kept in the neutral position, the validity of the linear regression model described is reduced.

Future prospects

Further research generating more data is required to construct a clinically applicable model for tendon-graft tensioning in the secondary repair of a jersey finger.

This can be achieved by further cadaveric study or through the examination of existing MRI scans of hands. This could then be followed by clinically applying the model and assessing functional outcomes and cascade restoration on a small sample group, with a view to conducting larger clinical trials.

Trials attempting to record measurements intra-operatively may consider issues regarding the biomechanical properties between live tissue and preserved specimens. Inquiry into the comparative biomechanical properties of native FDP tendons vs. graft tendons (e.g. elasticity) is also crucial, as differences in tendon elasticity may require a graft length adjustment to that advocated by the present study.

Our measurements were based on taking digit III as the basis. This means we are unable to estimate middle finger tendon segment lengths. Future work could focus on comparing the variability in measurements between patient’s hands. If these are found to have low variability, we could consider imaging the contralateral hand in order to establish the length of graft required.

## Conclusions

This study proposes a novel framework for estimating tendon graft lengths for the secondary repair of jersey finger injuries. This has been achieved using lumbrical origin to the FDP insertion lengths across digits II-IV to generate linear regression models with a high level of predictive accuracy. However, larger prospective studies using existing MR imaging, cadavers and live tissue are required to clarify this relationship and explore its clinical applications. Our findings are promising, and we believe the methodology outlined serves as a suitable starting point for additional data to be added.

## References

[1] Goodson A, Morgan M, Rajeswaran G, Lee J, Katsarma E (2010). Current management of jersey finger in rugby players: case series and literature review. Hand Surg.

[2] Bachoura A, Ferikes AJ, Lubahn JD (2017). A review of mallet finger and jersey finger injuries in the athlete. Curr Rev Musculoskelet Med.

[3] Leddy JP, Packer JW (1977). Avulsion of the profundus tendon insertion in athletes. J Hand Surg Am.

[4] Bynum DK Jr, Gilbert JA (1988). Avulsion of the flexor digitorum profundus: anatomic and biomechanical considerations. J Hand Surg Am.

[5] Yeoman TF, Rust PA (2016). A rugby player's finger injury. BMJ.

[6] Gaston A, Allavena C, Mansat P, Rongières M, Mansat M (2009). Traumatic avulsion of the flexor digitorum profundus tendon. Report of 20 cases [Article in French]. Chir Main.

[7] Wallmann HW (2011). Overview of wrist and hand orthopaedic special tests. Home Health Care Manag Pract.

[8] Lapegue F, Andre A, Brun C, Bakouche S, Chiavassa H, Sans N, Faruch M (2015). Traumatic flexor tendon injuries. Diagn Interv Imaging.

[9] Hallock GG (1994). The Mitek Mini GII anchor introduced for tendon reinsertion in the hand. Ann Plast Surg.

[10] Schannen A, Cohen-Tanugi S, Konigsberg M, Noback P, Strauch RJ (2017). A novel cadaveric model of the Quadriga effect. J Am Acad Orthop Surg Glob Res Rev.

[11] Bruner JM (1967). The zig-zag volar-digital incision for flexor-tendon surgery. Plast Reconstr Surg.

[12] Parminder K (2013). Morphological study of lumbricals - a cadaveric study. J Clin Diagn Res.

[13] Fiorini HJ, Santos JB, Hirakawa CK, Sato ES, Faloppa F, Albertoni WM (2011). Anatomical study of the A1 pulley: length and location by means of cutaneous landmarks on the palmar surface. J Hand Surg Am.

[14] Kapickis M (2009). New "loop" suture for FDP zone I injuries. Tech Hand Up Extrem Surg.

[15] Brenner E (2014). Human body preservation - old and new techniques. J Anat.

